# Chairman’s Address

**Published:** 1897-08

**Authors:** R. R. Andrews

**Affiliations:** Cambridge, Mass.


### Chairman's Address.

BY R. R. ANDREWS, M.D , D D.S., CAMBRIDGE, MASS.

Abstract of Paper read before the Dental Section American Medical Association, Philadelphia, June, 1897.

In the annual address delivered before this section at its last meeting, I gave the results of my investigations of the development and structure of the dental enamel and in a friendly way criticised some of the views of another writer who had recently written on the same subject. I have given considerable time to the further investigation of the subject, preparing a paper which I read before the New York Institute of Stomatology. This paper was discussed by some of the more prominent biologists of that city, who agreed substantially with me in every point presented to you at our last meeting. The only important point on this subject I have to present as new matter is the fact before unnoticed, that some of the globular bodies that have been deposited by the ameloblast to form the enamel rods are not homogeneous masses of substance, but are globules having within their substance aggregations of minute spheres which for some reason have not coalesced into a mass. These minute globular bodies are held together by what appears to be an envelope or membrane, the whole forming a globule of the same size of what I call the normal enamel globule at its point of calcification. In some of my sections I have found some of these enamel globules containing the minute spheres, covering quite an area of forming enamel, and they are shown in this condition as they are drawn down into the calcifying rod. They are more than likely calcified in this condition. It would appear as though it were a fault of structure, perhaps some arrest in the developmental process.

A very valuable contribution to the study of the Pathology of the Enamel, by Dr. J. Leon Williams, of London, has recently appeared in the *Dental Cosmos*. While the subject-matter of the text is not altogether new to those familiar with this line of investigation, the lucid way in which the subject is presented makes it the most notable contribution to the literature of this subject that has yet been presented to the dental profession. I can heartily agree with Dr. Williams in the conclusions advanced, and trust that we may have this work published in a more permanent form for reference and for the use of students in our educational institutions.

The year that has passed has pretty conclusively proven that the use of dental cataphoresis may no longer be considered as in its experimental stage, so far as obtunding sensitive dentine and bleaching are concerned. The method has been found certain in its action in fully obtunding exposed pulps prior to their removal.

Investigations are being made by this method which promises success in the treatment for the relief and cure of chronic cases of pericementitis by a perfect system of sterilizing root-canals at the time of filling. It has been found that almost any kind of good antiseptic can be used, oil of cassia, hydrogen dioxid, clorid of zinc, and silver nitrate have all been tried with success. The method of using being something like that used for obtunding sensitive dentine. This treatment is said to allay the pain in a short time and place the tooth at once in a condition for immediate root filling. The results obtained by using silver nitrate are significant. Sections made from teeth which had been treated by a solution of silver nitrate by Dr. L. P. Bethel, of Kent, Ohio, were sent to me for microscopical examination. The higher powers of the microscope revealed the fact that the silver salt had been forced, by the current, a very considerable distance into the dentinal canals, and each canal had been completely sealed by it. As the dentine of the root is almost always infected by a putrescent pulp, which sends its organisms into the canals of the root, we can clearly see the importance of this perfect method of sterilization.

The very considerable value of the X ray as an assistance in diagnosis in our specialty has been repeatedly demonstrated during the year that has passed. It is possible to make skiagraphs of any part of the mouth or jaws, showing the teeth in proper relation with their alveolar socket. Thus any peculiarity of the roots may be noted.

Within the past year there has been a movement on the part of some of the dentists of Baltimore to bring about the passage of a law that shall require the examination of the condition of the mouths and teeth of school children. The time of life that the school years cover is very important to the child from a dental standpoint. If the teeth are cared for during this period, the chances favor their permanent retention, and for that reason, if no other, this movement should be urged and encouraged by every intelligent dentist. The matter is of such vital importance that we should seek the co-operation of every dental society in the land to aid us in our efforts to bring about the examination and treatment of the mouths of children in our public schools, who better than they can recognize the justice and importance of this matter. It will save the children from sorrow and suffering, and, in many cases, such supervision will triumph over disease and death.

CARIOUS TEETH AND TUBERCULOUS CERVICAL GLANDS.

Dr. Stark, in the *Revue de la Tuberculese*, notesthe frequent association between carious teeth and enlargement of the cervical lymph glands. He examined 113 children with enlargement of cervical glands, and, not being able to find other apparent cause for the condition, concluded that the swollen glands resulted from defective teeth in a series of cases. They corresponded in situation, time of development and degree of enlargement with the condition of the teeth.

The involved glands were situated on the same side as the diseased teeth, the anterior glands being enlarged if the incisors were carious and those at the angle of the jaw when the molars were involved. Toothache frequently preceded the enlargement of the glands. Dr. Stark is of the opinion that the enlarged glands are tuberculous in quite a number of these cases. He cites two cases in which he was able to demonstrate pretty conclusively that the carious teeth had been the point of entrance of the tubercle bacillus.

Case 1 was a boy, age thirteen, who had always been healthy and without a history of tuberculosis, had caries of the molar teeth on both sides with enlargement of the cervical glands. The glands were excised and the teeth extracted. The former proved to be tuberculous, and tubercle bacilla were found on cover-slip preparations from two of the decayed teeth.

Case 2 was a girl, age fourteen, with excellent personal history, and without a history of tuberculosis in the family. The first inferior left molar was carious and there was an enlarged gland below the ramus of the jaw. The gland was removed and showed definite tubercular disease. Between the diseased molar and the adjacent tooth, and also forming the floor of the cavity in the tooth, were characteristic tuberculous nodules with typical giant cells. The latter case is particularly interesting as it seems to show that a carious tooth may be the seat of a primary tuberculous focus. With regard to treatment, the glands should at once be removed and the carious teeth should be either properly filled or extracted. As a prophylactic, the teeth should be better cared for, and Dr. Stark, with Dr. Rose, advocates the placing of all small school children under supervision.

Another interesting case has just been reported to me by a brother dentist. A boy, age sixteen, who was an invalid, had teeth so extremely sensitive that he could not, or would not, have them filled when they decayed. He had trouble with his cervical glands and had them operated upon, with relief for a number of years. Afterwards it was found that other glands were infected with tuberculous disease and the patient was taken to New York for treatment. The surgeon who performed the operation of excising the infected glands insisted that all of the decayed teeth in the patient's mouth should be at once removed, giving as his reason that the decayed teeth were largely the cause of the infection.

